# Anthropogenic Land Use and Land Cover Change as Potential Drivers of Sediment Sources in the Upper Crocodile River, North West Province, South Africa

**DOI:** 10.3390/ijerph192013313

**Published:** 2022-10-15

**Authors:** Samuel Che Nde, Sammy Kipyego Bett, Manny Mathuthu, Lobina Palamuleni

**Affiliations:** 1 Unit of Environmental Science and Management, Faculty of Natural and Agricultural Sciences, North-West University (Mahikeng Campus), Mmabatho 2735, South Africa; 2 Department of Geography and Environmental Sciences, North-West University (Mahikeng Campus), Mmabatho 2735, South Africa; 3 Centre for Applied Radiation Science and Technology, North-West University (Mahikeng Campus), Mmabatho 2735, South Africa

**Keywords:** land use/land cover dynamic, sediment contamination, river pollution, sediment source tracing

## Abstract

In this study, we investigated the accelerating pace of anthropogenic land use and land cover change (LULCC) disturbance, which has generated enormous impacts on the Crocodile River. Spot images from 1996, 2009 and 2022 were used to generate the land use maps and quantify the changes. A supervised classification with the maximum likelihood classifier was used to classify the images. Sediment sources were classified into two sources, revealed by erosional characteristics in the catchment. A gamma spectrometry detector, high-purity germanium (HPGe) “Well” detector by Canberra and inductively coupled plasma mass spectrometry (ICP–MS) were used for the analysis of the samples. The results revealed that from 1996–2022, built-up areas, bare land and water bodies increased by 3.48%, 2.47% and 1.90%, respectively. All the LULCC classes increased annually from 1996–2022, except for grassland, which shrunk. The results of the radionuclides analysis showed that ^210^Pb_ex_ was found to be a more effective tracer than ^137^Cs. The mass balance model revealed that subsurface sources contributed 60%, while surface sources contributed 40%, of the sediment load in the river. This research provides valuable information necessary for integrated catchment management policies for future LULCC and soil erosion to be adopted.

## 1. Introduction

Rivers play a vital role in sustaining marine organisms and human populations that directly and indirectly depend on it [1]. However, the progressive deterioration of rivers has resulted from a regional and global crisis, accentuated by increased climate change [2,3]. The United Nations (UN) World Water Department Report projects that the strain on rivers will have grown by 22 to 34% by 2050 [4]. Boretti and Rosa [5] reiterate that the global water demand will increase significantly in the next few decades in all the three components, including agriculture, industry and domestic usage. This situation is likely to constrain the rivers in their roles as regulators of the landscape and moisture redistribution and in maintaining biological diversity and economic operations [6]. Agricultural intensification, mining operations, urbanization, increased construction levels and rapid population growth are major contemporary anthropogenic drivers that have led to irreversible changes in landscape structures, which generate enormous impacts on rivers, causing the degradation of aquatic ecosystems and habitats [7]. Considering this threat to rivers, there exists an urgent need for information that can help us to better understand and recognise human impacts and their negative effects on the landscape.

Land use and land cover change (LULCC) contribute to land fragmentation, amplified by increased human settlement through the alteration of the natural landscape [8,9,10]. This already dire situation is further exacerbated by changes in land use patterns, such as the conversion of grassland to paddies, woodland to cropland and bare land to built-up areas, which inevitably accelerate surface water pollution and hydrological connectivity, leading to sediment delivery in the river systems [11,12]. According to Aneseyee et al. [13], LULCC are major causal drivers of soil loss in upland watersheds, indicating the transformative interaction between land use changes and land degradation. Different studies [9,14,15,16,17] have investigated the association between LULCC, soil erosion and river contamination. However, the analysis of LULCC as causal drivers of land degradation alone cannot quantitatively distinguish the relative contributions of each type of sediment source to the overall sediment load being delivered into rivers. Thus, the utility of the sediment source fingerprinting technique for generating valuable information on the relative individual potential sources contributing to downstream sediment has become apparent [18,19,20,21,22,23,24]. To date, studies that have used the fingerprinting technique to trace sources of sediment and its accompanying fluvial materials have been insightful, particularly in distinguishing between surface and subsurface erosion sources [25,26]. Most existing studies have used ^137^Cs and ^210^Pb_ex_, or both in combination, for dating sediment or tracing the sources of sediment. As an anthropogenic isotope, ^137^Cs was introduced into the atmosphere during the testing of thermonuclear weapons in the 1950s, while ^210^Pb_ex_ is a product of the uranium-238 (^238^U) decay series. These isotopes have been proven to have a strong affinity for soil and sediment particles and are thus used in erosion studies and sediment source tracing [27,28,29]. The authors quantitatively traced the contributions of sediment sources, revealed the impacts of anthropogenic activities on erosion and recommended the best management practice with respect to surface soil erosion control measures. Additionally, Du and Walling [30] and Zhang et al. [31] reiterate that sediment fingerprinting is an evolving research tool, which needs further refinement. This assertion implies that that there is a need to further expand the scope of fingerprinting research, especially in areas where such studies have never been conducted before, as is the case for the Upper Crocodile River catchment.

GIS and remote-sensing-based studies have played a significant role in geographical mapping, the monitoring of land use and land cover and change detection [32,33]. These techniques have been accepted as global research tools and have been widely used to generate valuable quantifiable data, enabling researchers to visualise present and future changes in different environments [34,35,36]. Different scholars have used the maximum likelihood classifier (MLC) [37,38], artificial neural network (ANN) [39], CA–Markov model and built-in GIS software and algorithms [15,40] to quantitatively and qualitatively analyse change detection and predict future scenarios using maps. However, there are limited studies which have applied the fingerprinting technique by incorporating LULCC in assessing environmental changes. Unlike other geospatial models, the incorporation of the fingerprinting technique, alongside geospatial analyses, is very useful in studies of this kind, as these methods provide a quantitative estimation of the associated LULCC and reveal its negative environmental effects.

**Figure 1:**
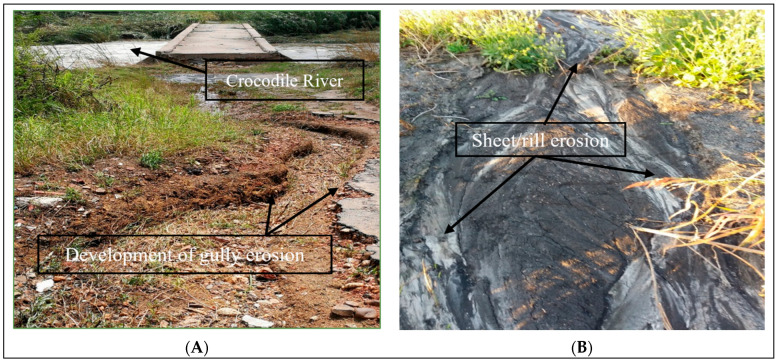
An illustration of an eroding field draining into the Crocodile River: (**A**) shows a developing gully erosion and (**B**) shows evidence of sheet/rill erosion draining into the Crocodile River.

Of particular interest to this study is the fact that semi-arid regions face water shortages and severe soil erosion, which is irreversible [31]. The North West province of South Africa, being a semi-arid province, is no different from other semi-arid areas facing such a plethora of environmental problems. Different studies have reported that approximately 70% of South Africa’s surface is affected by varying intensities and types of erosion [26,41,42,43]. This has led to an increased sediment yield, resulting in soil losses that often exceed 150 t ha^−1^-y^−1^ and accumulate in river systems and farm dams. The Upper Crocodile River Catchment has undergone decades of intensive arable land use and mining activities and, more recently, rapid urbanisation, which has resulted in significant landscape reconfiguration [44,45]. Increased field sizes for cultivation, on-field stocking densities and artificial canal construction for irrigation and drainage systems have had great impacts on the different land use and land cover classes, thereby enhancing the potential of soil erosion caused by diverse types of land use to drain into the Crocodile River [46]. It is clear that river ecosystems in South Africa are at risk, with an uncertain and potentially negative future if nothing is done. Evidence gathered from the field survey revealed surface and sub-surface soil erosion causing severe damage by increasing the connectivity and the transfer of sediments to the Crocodile River (Figure 1). This already vulnerable region has an extremely sensitive environment, which makes it significant for the analysis of anthropogenically induced land use and land cover changes as sources of surface and subsurface soil erosion polluting the Crocodile River. If proper mitigation measures are to be adopted effectively and efficiently, there is, first, a need to identify the main sediment sources polluting the river, which will be useful in enabling us to further understand the erosion processes caused by the anthropogenic LULCC.

At present, there is no information to indicate how anthropogenic land use change patterns influence sediment delivery into the Crocodile River. This information is required in order to develop proper mitigation measures in the catchment and to predict future sediment yield changes that might arise due to LULCC. The key objectives of this study were (1) to analyse the main land use changes through the evaluation of the relationship between the LULCC causing land degradation along the Crocodile River and (2) to spatially identify the main erosion characteristic revealed by the quantification of the surface and subsurface sources. The results of this study will be useful for designing proper management and control strategies for erosion caused by increased multiple anthropogenic activities in the catchment, while also adding to the general improvement of the use of multiple fingerprinting signatures. Additionally, this study contributes to the fingerprinting literature by extending it so as to include LULCC and environmental monitoring and management.

## 2. Materials and Methods

### 2.1. Study Area

**Figure 2:**
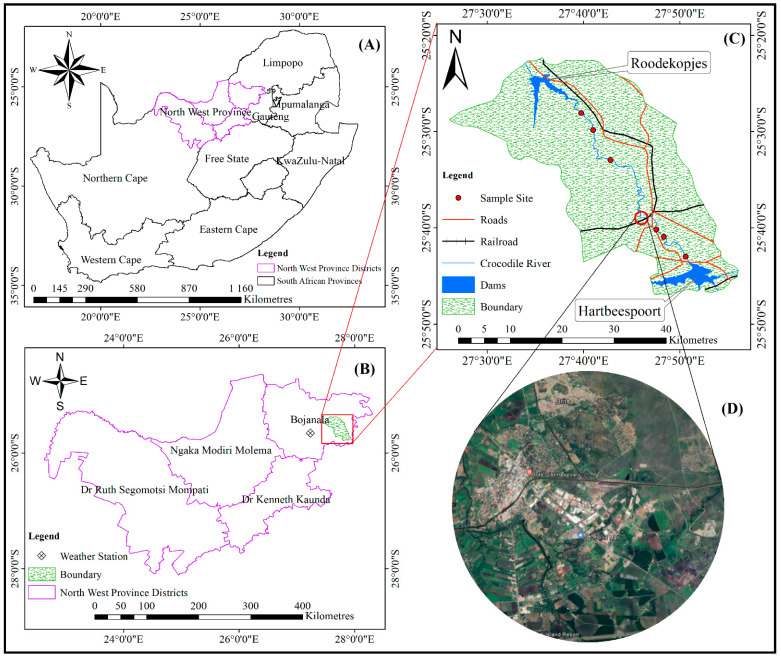
Map of the study area: (**A**) shows the map of South Africa, (**B**) shows the different provinces in South Africa, (**C**) shows the study catchment area and (**D**) shows a zoomed-out area of the different land uses.

The study area is located in the Upper Crocodile River catchment, (Figure 2). The study catchment is located in Rustenburg, which is the economic hub of the North-West Province (Figure 2B). The studied catchment is approximately 168,777 km^2^, incorporating the Crocodile River, which flows in a northward direction. The area forms part of the Upper Crocodile River Catchment, with its main tributaries being the Hex, Elands and Sterkstroom rivers, as well as the Jukskei and Hennops Rivers and other non-perennial streams [47]. The sub-catchment has two major dams (Hartbeespoort and Roodekopjes), with dams scattered throughout the catchment (Figure 2C). The fluvial sediment in the Crocodile River varies from boulders with a diameter of 2.5 m to clay-grade sediments. The catchment area contains flood channels, bars and terraces comprised of mainly sandy-grade materials, which can ingress into the gravel riverbed [48]. The predominant soil texture of the investigated area includes sandy clay loam, clay and clay loam [49].

**Figure 3:**
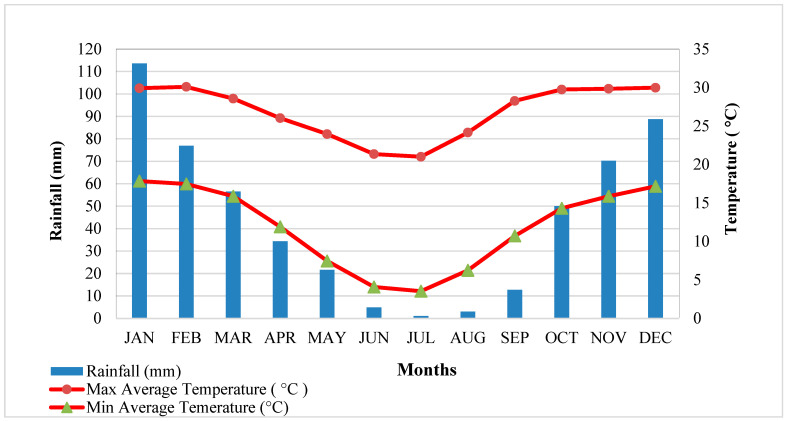
Rainfall and temperature averages for Rustenburg (1993–2016).

The underlying geological rock structure of the area is dominated by the Pretoria Group, part of the Transvaal Supergroup, and lies on the edge of the Bushveld Igneous Complex [47], which is one of the most heavily mineralised and diverse mineral-producing districts in the world (platinum group of metals). The formations of this complex are extremely rich in diverse minerals, and a number of mines have been developed in the area, with several decades of intensive mining for chrome, vanadium, tin, lead, marble and granite, among other materials [44]. The resultant effect of the mining operations has led to an influx of skilled and unskilled labourers from the surrounding villages, coupled with urbanisation and industrial expansion, as well as diverse forms of recreational facilities and crop cultivation along the banks of Crocodile River [50,51]. The common land use types in the studied catchment are cropland, grassland, bare land, built-up areas and water bodies comprised of natural flowing rivers and artificial dams and canals. Figure 2D shows a zoomed-out satellite image depicting the different land use types, but it does not show the entire catchment and the sampling points, due to the map resolution and the map scale, but these are well explained herein. The climatic conditions of the region vary seasonally, constituting a dry subtropical climate with convectional summer rain [47]. Most rainfall occurs during the summer period from October to March and can extend to April, and the months from June to August and sometimes September produce the lowest average monthly rainfall (Figure 3). The monthly minimum and maximum average temperatures range from 3 °C to 7 °C from May to August, being the coldest months in winter, and vary between 14 °C and 30 °C in summer, between October and February (Figure 3).

### 2.2. LULC Data Sources

This study used geospatial and remote sensing data sources, since these provide first-hand information that is useful for identifying and understanding the dynamics and drivers of the LULCC of any landscape [15,46]. Remote-sensed data from 1996, 2009 and 2022 were selected for this study. The data were obtained from the South African National Space Agency (SANSA) archives. SPOT images were preferred in this study due to their high spatial resolution and ability to identify changes in land use patterns (Table 1). Because of differences in radiometric resolution (Table 1), the image data were converted from digital numbers (DN) to at-sensor radiance (L_SAT_) units (W m^−2^ sr^−1^ μm^−1^). The chosen dates of the images fell between April and May, as they offer the benefits of clear skies, consistency in cover classes and phenology.

#### 2.2.1. Data Pre-Processing

Relative geometric corrections were performed using the three images in order to remove geometric distortions and because the images had different spatial resolutions [52,53]. This measurement was performed using the root-mean-square error (RMSE) to obtain pixel sizes of a common value. The satellite imagery was geo-referenced using ground control points (GCPs) and projected using the Universal Transverse Mercator (UTM) system projection. Subsequently, the geo-referenced terrain was used to verify that the land use and land cover class map passed the test of the ground-truth method, as prescribed by Daba and You [15]. A combination of ground-truth validation, Google Earth^®^ imagery and a visually interpreted detailed topographic map with a scale of 1:50,000 produced in 1996 were used to delineate and identify sample training locations. The image produced in 1996 was used to geocode the image from 2009 and used to register the images from 1999 and 2022. For the three images used, the acceptable RMSE pixel was less than 0.4 [54]. A multiresolution segmentation algorithm was used to conduct the image segmentation [55]. The weights of the band, the shape (and its corresponding colour), the scale parameter and the compactness (and its corresponding smoothness) were specified to the algorithm, as outlined by Benz et al. [56].

#### 2.2.2. Analysis of the LULC Change and Accuracy Assessments

In order to determine the anthropogenic LULCC of the study catchment, the maximum likelihood classifier (MLC) method was adopted, as expounded in a study by Che, Bett, Okpara, Olagbaju, Fayemi and Mathuthu [46]. The accuracy assessment was performed following the procedures outlined by Mariye, Mariyo, Changming, Teffera and Weldegebrial [14], comparing the classified image with the land cover classes on the topographic map and the ground control points on the field. The area was classified into five broad LULC categories, namely, cropland, grassland, bare land, built-up areas and water bodies, with their descriptions given in Table 2. By defining a signature file and assigning the number of land use and land cover classes, ERDAS Imagine and eCognition Developer 9 were used to compute the land use and land cover classifications through a supervised classification method (i.e., maximum likelihood classifier). The different land cover classes were generated by creating 200 random points of the study area per image datum using a random stratified method. The “precision points” function in ERDAS Imagine 2020 was used for the MLC classified images to generate a set of random points. The reference data against which we judged the correctness of the classifications were obtained from 10 m resolution images on Google Earth^®^ taken on dates close to those of the SPOT images. The rate of change for each land use and land cover class over time (percentages) was calculated using Equations (1) and (2), modified from Aneseyee, Elias, Soromessa and Feyisa [13] and Dibaba, Demissie and Miegel [57].

(1) $$Total\;LULC\;gain/loss=A_{t2}-A_{t1}$$

(2) $$C=\frac{A_{t2}-A_{t1}}{A^{1}}\times 100$$

where *A_t_*_1_ is the area of one type of land use in time *t*1; *A_t_*_2_ is the area of the same type in *t*2; *A*^1^ is the total area of the catchment; and *C* is the percentage of LULCC gain/loss.

An accuracy assessment, also known as the confusion matrix (error matrix), of all the classified images was conducted to evaluate the user’s accuracy (UA), producer’s accuracy (PA), the Kappa coefficient ($\hat{K}$) and the overall accuracy (OA) using all the classified SPOT images [58]. The matrix was used to compare the ground truth data obtained from the reference sites with the classified image results of the sample areas, as outlined by Aneseyee, Elias, Soromessa and Feyisa [13]. The results of the calculation of the confusion matrix are presented in Table 3.

### 2.3. Soil Sampling

Consistent with other reported sediment source tracing studies [25,26,42], a longitudinal transect was adopted, considering the land use types, within the profile of the river in order to identify potential sediment sources and sinks. This was to ensure that each sampling point reflected the different land uses along the Crocodile River, and where there was no clear distinction of the land use type, both land use types were combined [51]. Two potential sediment sources were identified based on the relatively severe erosion in the study area observed in the field survey (Figure 1A,B). All the surface samples (n = 27) were collected on the 18 May 2017 from eroding areas at a depth of 0–2 cm. Sediment grab samples (n = 8) were collected on the 14 June 2017, resulting from a recently deposited instream of the main river during periods of low flow (dry season). Additionally, sediment samples (n = 8) were collected on the 28 August 2017, at the place where the stream enters the Hartbeespoort dam, by surficial scarping of the deposited sediment layers using plastic trowels during the dry season, when the river flow has receded to represent the sink areas. All subsurface sources (n = 34) were collected from recently developed gully walls (Figure 1A), while the riverbank samples were collected at a depth below 20 cm, as prescribed by [26,59]. Both types of samples were collected on the 5 April 2018.

Fine-grained riverbed sediment samples (n = 8) that showed signs of recent deposition were collected on the 10 April 2018 at channel sites to compensate for missing sediments due to high flows or poor access to the river [24]. Other studies [60,61] have reported that riverbed fine-grained sediment samples that appeared to have been recently deposited, referred to as “drape”, can be used as an alternative to sediment traps. A total of (n = 85) soil samples were collected, and all the samples were packed into labelled plastic bags and taken to the laboratory for analysis. The samples’ geographical coordinates were recorded using a portable handheld global positioning system (GPS) produced by Garmin.

### 2.4. Laboratory Analysis

#### 2.4.1. Gamma Spectrometry Analysis

In the laboratory, all the samples were oven-dried at a temperature of 60 °C and then gently disaggregated with a non-metal mortar and pestle. For the gamma spectrometry analysis, the soil samples were sieved in order to collect < 2 mm- diameter fractions, since ^137^Cs and unsupported ^210^Pb are known to be absorbed by soil particles of sizes under 2 mm [26,62]. Prior to the measurements, each vial was weight, washed and sterilised with distilled water and then dried. The samples were subsequently packed into 7 cm- and 9 mm-diameter vials, with the soil samples at a depth of 4 cm so as to match the geometry of the high-purity germanium (HPGe) “Well” detector gamma spectrometry by Canberra. The energy of the equipment was calibrated using gamma-certified reference sources provided by the International Atomic Energy Agency (IAEA), while the efficiency was calibrated using the multiple radionuclide sediments from the Irish Sea (IAEA-385). The samples were re-weighed after sealing with a rubber seal and paraffin wax to prevent the escape of ^222^Rn gas. The sealed vials were kept for thirty-one (31) days to reach the secular equilibrium between the in situ ^226^Ra and its daughter ^222^Rn. The counting times were typically 259,200 s, on average, per vial. The activity concentrations of ^137^Cs and ^210^Pb_ex_ were measured at different resolutions (FWHM), of which that at 122 keV ((57Co) is 0.85 keV and that at 1332.5 keV (60Co) is 1.86 keV, and the relative efficiency for the energy of 1.33 MeV relative to (NaITl) is 36%. The activity per sample was obtained using the selected radionuclide with the Genie 2000 Gamma analysis software.

#### 2.4.2. Geochemical Analysis

Similar to the gamma spectrometry analysis, all the source and sediment samples were sieved to obtain <63 μm samples and then measured using inductively coupled plasma mass spectrometry (ICP-MS) (Perkin Elmer Nixon 300Q) [31]. A Microwave Multiwave 3000, Anton Paar, Aqua-regia digestion method was used, where we used a 1 g aliquot of the sample using an acid mixture of 70% hypochlorite acid, 69% nitric acid and 30% hydrogen peroxide, as prescribed by [63]. The geochemical elements included Be, B, Na, Mg, Al, P, K, Ca, Ti, V, Cr, Mn, Fe, Co, Ni, Cu, Zn, As, Se, Rb, Sr, Mo, Pd, Ag, Cd, Sb, Ba, Pt, Pb, Bi, Th and U. The equipment was calibrated following similar procedures outlined by [63].

#### 2.4.3. Sediment Source Ascription and Modelling

Different statistical tests were used prior to selecting the fingerprinting properties that could best discriminate between the two potential sediment sources with less uncertainty [18]. This involved testing fingerprinting dataset for normality for each of the source categories by employing the Shapiro–Wilk test, specifically designed to test the normality of any sample distribution. The Shapiro–Wilk test suggested the absence of normality (*p* < 0.05) for both the surface and subsurface soil samples [23]. Nosrati, Fathi and Collins [20] suggests that different combinations of statistical tests could be further explored when selecting the optimum fingerprinting properties which can best discriminate between the potential sediment sources. In line with the above mentioned assertion, Dunn’s post hoc test was carried out on each pair of fingerprinting groups, in which adjustment to the *p*-value was performed using the Bonferroni error correction to adjust for concurrent multiple comparison tests associated with an increase in the error.

The multivariate mixing model was used to estimate the proportions of each potential sediment source, as prescribed by Manjoro, Rowntree, Kakembo, Foster and Collins [25] and Miller et al. [64], in order to estimate the sources sediment flowing from the surface and subsurface to the river, using Equation (3):

(3) $$\overset{n}{\underset{i=1}{\sum }}{\left\{\frac{\left(C_{i}-\left(\sum_{s=1}^{m}\;P_{s}S_{si}\right)\right)}{C_{i}}\right\}}^{2}$$

where *n* is the number of tracer properties of the optimum composite tracer, *C_i_* is the concentration of the *i*th tracer property in the sediment, which is the optimised percentage contribution of source *s*, *Ss_i_,* is the mean concentration of the *i*th tracer property in source *S* and *m* is the number of sediment sources. Constraints on the mixing model ensured that (1) the contributions of the individual source areas were non-negative $\left(0\leq P_{i}\leq 1\right)$ and (2) the sum of the relative contributions from all of the source areas was equal to unity (Equation (4)) [64].

(4) $$\overset{n}{\underset{s=1}{\sum }}{Ρ}_{s}=1$$

#### 2.4.4. Quality Control/Quality Assurance

In this study, quality control/quality assurance is highly important, and it has been established by similar studies, which are referenced herein [26,46].

## 3. Results and Discussions

### 3.1. Accuracy Assessment

The results of the confusion matrix and Kappa statistics used to test the classification accuracy of the land use and land cover maps for the periods of 1996, 2009 and 2022 are presented in Table 3. The overall accuracy assessment results for the 1996, 2009 and 2022 maps were 76%, 82% and 85%, respectively. The Kappa statistics were 0.75, 0.81 and 0.83 for the maps of 1996, 2009 and 2022, respectively, thus being in good agreement with the other study [65].

### 3.2. Spatio-Temporal Distributions of LULC Changes in the Crocodile River Catchment

**Figure 4:**
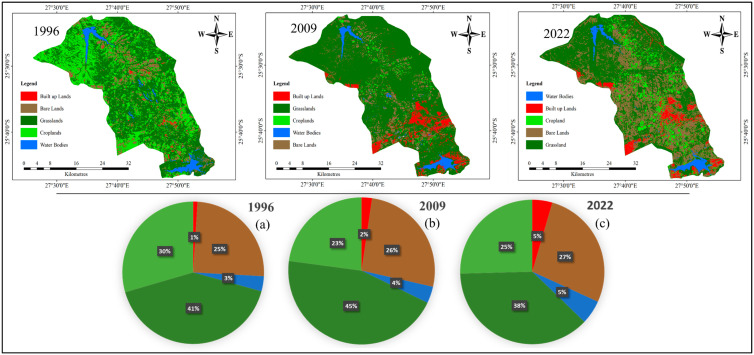
Land use distribution and percentage of areas of different land use types to the total basin areas in 1996 (**a**), 2009 (**b**) and 2022 (**c**).

The results of LULC analysis indicate significant changes in the spatiotemporal transformation of the catchment area during the study period (Figure 4 and Table 4A,B). The proportion of built-up areas observed in 1996, 2009 and 2022 to the total land cover area shows an increasing trend from 1% to 2% and 5%, respectively (Figure 4a–c). Similarly, water bodies also increased in proportion to the total area from 3% to 4% and 5%, respectively, from 1996 to 2009 and 2022. Bare land covered 25%, 26% and 27% of the catchment in 1996, 2009 and 2022, respectively. Grassland accounted for 41% (1996), 45% (2009) and 37% (2022), and cropland accounted for 30% (1996), 23% (2009) and 25% (2022). The area used for cropland shrunk from 49, 758 ha in 1996 to 38, 629 ha in 2009 but increased to 42, 897 ha in 2022.

The distribution of the land cover over two decades, as shown in Table 4A, indicates a continuous increase in, and expansion of, built-up areas, water bodies and bare land. This expansion is linked to accelerated human developmental activities implemented in the study area, which have led to the complex transformation of the catchment landscape. As the population increases, there is need to expand the road constructions and housing and increase the number of irrigation systems for crop cultivation, which in turn will lead to the creation of more artificial dams and canals for irrigation purposes. As such, the catchment area has experienced an expansion of water bodies and built-up areas. A study by Daba and You [15] found that changes in land use and the land cover transformation of the Awash river basin, which is the second-largest river in Ethiopia, was mainly triggered by population increase. Additionally, the study by Dibaba, Demissie and Miegel [57] showed that urban expansion was the main driver of deforestation and land conversion for housing and other infrastructure developments. As shown by the remote-sensing LULCC analysis of the past 26 years (1996–2022), built-up areas, bare land and water bodies in the lower reaches of the Crocodile River basin have increased significantly in the area close to the Hartbeespoort dam (Figure 4). Different studies have highlighted the observation of algae and cyanobacteria booms in the Hartbeespoort dams, mainly due to changes in land use and land cover [66]. Changes in the use of croplands, built-up areas and bare land might be the influential activity leading to increased nutrient input, particle-borne contamination and heavy metal transport from untreated wastewater effluents and construction, deteriorating the surface water quality of the Hartbeespoort dam [47].

The highest increase occurred in built-up areas, followed by bare land and water bodies, whereas cropland had the highest loss, followed by grassland (Table 4A). The annual rate of change for all the land use and land cover classes increased from 1996–2009, except for cropland, which showed a declining trend by −0.51% but increased from 2009–2022 (Table 4B). From 2009 to 2022, built-up area, bare land, water bodies and cropland showed an increasing annual trajectory, whereas grassland shrunk by −0.58%. This result shows that from 2009 to 2022, more land conversion occurred. This conversion might have been triggered by various intermingling factors, such as urban expansion, demographic factors, agricultural expansion and biophysical factors in the catchment. The land use and land cover of the built-up areas, bare land, cropland and water bodies underwent tremendous changes, exerting pressure on non-urban areas, especially those along the Crocodile River towards the Hartbeespoort dam and downstream. Through the construction of residential private resorts and holiday homes, commercial units, road networks, sidewalks and ports for different commercial boats operating around the dam, it is plausible that this increase might have contributed further to the degradation of the pristine environment [46].

### 3.3. Sediment Source Fingerprinting

#### 3.3.1. Environmental Radionuclide and Geochemical Fingerprints

The results of the analysis of the activity concentrations of ^210^Pb_ex_ and ^137^Cs are presented in Table 5. The results indicate that ^137^Cs was not found in any of the soil samples analysed. The lack of ^137^Cs in both the surface and subsurface soil samples for all the land use types in the study catchment could be due to radioactive decay and low fallout in the Southern Hemisphere [67]. Other studies have equally reported on the absence or low activity concentration of ^137^Cs in countries in the Southern Hemisphere, attributed to low activity fallout as compared to that in the Northern Hemisphere [68]. The findings of this study further corroborate those of Nde, Manjoro and Mathuthu [26], who reported the absence of ^137^Cs in the North West Province of South Africa. On the other hand, ^210^Pb_ex_ showed higher activity concentrations in the surface samples than in the subsurface samples (Table 5).

The agriculture and mining land use concentrations of ^210^Pb_ex_ in the surface soil samples were both around 10.01 ± 01 Bq kg^−1^, while the subsurface soil samples values were all below the detection limits. The resort/commercial land use values for the surface soil samples were significantly higher (140 ± 9 Bq kg^−1^) than those of the subsurface samples (63 ± 32 Bq kg^−1^). The low activity concentrations in the case of agricultural land use might be due to constant ploughing with tractors during the farming operations. It is possible that at the time of the sample collection, most of the ^210^Pb_ex_ deposited in the surface soil had been replaced by the subsurface samples, having little or no detectable ^210^Pb_ex_, or that these were buried deeper, since only the first 0–20 cm area was sampled. A study by Yang and Appleby [69] reported that the activity concentration of ^210^Pb_ex_ in soils declined rapidly from a depth of 4 cm to a point where its activity was effectively zero, below 8 cm in undisturbed soils. Similarly, the study by Caitcheon et al. [70] demonstrated that the ^210^Pb_ex_ activity, or concentration, in the surface tends to be significantly higher than that of the subsurface soil. The presence of detectable ^210^Pb_ex_ at a depth 0–3 cm in deposited sediment could be interpreted as an indication that the sediment was probably mobilised in the surface soil sources and, therefore, represents surface erosion, such as rill and/or sheet acting on the surface [71]. In contrast, deposited sediment with an undetectable or low activity concentration might indicate a subsurface soil source and is most likely to have been mobilised by gully and riverbank erosion, which might be influenced by changes in land use and land cover in the catchment.

The results of the geochemical normality tests of both the surface and subsurface samples are presented in Table 6. The results of the normality distribution of the fingerprinting properties suggest that not all the tracer (fingerprint) properties of both the surface and subsurface soils were normally distributed (Table 6). The result indicated that only the properties Mg, Ca, Al and Fe, which were associated according to the pairwise multiple comparisons and were statistically significant at (*p* < 0.05), should be chosen for further analysis, as presented in Table 7. The remaining 29 elements (Be, B, Na, P, K, Ti, V, Cr, Mn, Co, Ni, Cu, Zn, As, Se, Rb, Sr, Mo, Pd, Ag, Cd, Sb, Ba, Pt, Ti, Pb, Bi, Th and U) that did not pass the test were removed from the further analysis. It should, however, be noted that the selection of the tracer properties for the mass balance model was constrained by the available analytical data (i.e., environmental radionuclides and geochemical elements) and the purpose of the work under investigation.

#### 3.3.2. Estimates of the Sediment Contributions of the Surface and Subsurface Sources

The results of the mass balance model suggest that the subsurface soil sources contributed 60% of the sediment, while surface sources contributed 40% of the sediment flowing to the Crocodile River. The total contribution of all the sources must be summed up to 100%, because each contributing source represents the relative percentage, resulting in a wane and wax relationship of each contributing source [31]. Therefore, when one of the sources changes significantly, the other source will vary dependently. The subsurface was the main contributing source in this study. Thus, the variation in the contribution of the surface soil exhibited a certain dependency on the contribution of the subsurface. Other studies have equally reported that gully erosion is increasingly becoming a severe threat, leading to land degradation in South Africa [25,72]. It should, however, be noted that sediment delivery is a complex and dynamic process that can be caused by many cooperative processes, ranging from catchment sensibility and nature to the spatial distribution of rainfall.

Semi-arid zones are prone to increasing temperatures, prolonged droughts and excessive rainfall, which makes the land use and land cover of an area susceptible to different types of land degradation [15]. The historical fluctuations in rainfall and temperature (Figure 3) in the catchment are attributable to the considerable effects of climate change, causing prolonged drought/excessive rainfall and making the areas susceptible to different types of surface and subsurface erosion due to damaged soil structures during rainstorm events and dry spells. Evidence gathered during the field reconnaissance (Figure 1A,B) shows the development of rill and gully erosion, draining into the Crocodile River due to excessive rainfall over a short period of time. Anthropogenically induced land use changes have been recognized as the main factor influencing sediment delivery into rivers. For example, a study by Collins, Zhang, McChesney, Walling, Haley and Smith [23] reported that, in an agricultural watershed in southern England, subsurface sources of sediment were second to non-metal surface farm tracks as the most important sources of sediments in the river. Similarly, Stander, Le Roux, Abd Elbasit and Liu [68] demonstrated that soil erodibility represents the dominant soil factor in South Africa. Additionally, one possible explanation for the high contribution of the subsurface soil could be the fact that most of the cultivated areas act as production zones of more sediment due to the constant tilling of surfaces with less vegetation cover to act as a soil buffer. This result is consistent with the findings of authors of [58], who demonstrated that land use changes result in corresponding changes in the soil erosion rates and sediment export. When land use types are converted or changed, the spill-over effect leads to changes in hydrological processes, such as surface runoff, sediment production, evaporation and lateral flow [73,74].

The increase in land use and land cover components driven by anthropogenic activities has led to the gradual deterioration of water quality of the Crocodile River [51], as well as the degradation of flora, soil and fauna, leading to desertification through the expansion of bare land, which in turn leads to soil loss and the loss of biodiversity [75]. It has been noted that changes in land use and land cover aggravate the problems relating to the use of natural resources, especially water resources, in semi-arid areas [76], with the underlying factors related to increased human activities. The results of this study highlight the severity of soil erosion and sediment export aggravated by LULCC over the past 26 years, leading to soil loss and sediment export into the Crocodile River.

## 4. Conclusions

This study investigated anthropogenic changes associated with LULUC and the potential sources of sediments in the Upper Crocodile River catchment. The spatio-temporal analysis, through the interpretation of satellite images, shows that the Upper Crocodile River has undergone significant LULCC from 1996 to 2022. Between 1996 and 2022, built-up areas, water bodies and bare land increased, while grassland increased between 1996 and 2009 but decreased from 2009 to 2022. Cropland, on the other hand, decreased from 1996 to 2009 but increased from 2009 to 2022. The rate of change for all the land use and land cover classes increased annually from 1996 to 2009, except for cropland, which showed a decline, indicating a loss, but then increased from 2009 to 2022. The gamma spectrometry analysis indicates the absence of ^137^Cs in all the sediment and soil samples, thereby confirming its inability to be used as a tracer in any future sediment source investigations due to radioactive decay. The activity concentrations of ^210^Pb_ex_ in the surface samples were higher than those in the subsurface samples, indicating the possibility of rill/sheet erosion. The statistical analysis of the geochemical fingerprint properties showed that only Mg, Ca, Al and Fe satisfied the initial conditions that were used for the multivariate mixing model. The results of the mass balance model showed that the subsurface sources were the primary sediment sources, contributing 60% of the sediment, while the surface sediment sources contributed 40% of the sediment flowing into the river.

The adverse impacts of LULCC along the Crocodile River reveal human activities as the underlying causes of river pollution through sediment. Soil conservation measures are therefore paramount to the recovery of the environment through subsurface structures, according to which mitigation measures should be applied, especially along the Crocodile River, to achieve acceptable levels of soil erosion control. Some of the uncertainty regarding the use of the fingerprinting approach in this study will be more prominent in the case of complex catchments with different land use types acting as sediment source production zones, with respect to the sample screening for the selection of the optimum fingerprinting properties, the rationality of the source classification resulting from different land uses and the use of robust mixing models. It is therefore necessary to improve the fingerprint approach associated with LULCC and soil erosion in future research on complex environments.

## Figures and Tables

**Table 1:** List of satellite images collected for the study area.

Acquisition Date	Satellite	Sensor	Cloud Cover	Path (K)	Row (J)	Spatial Resolution		Pre-Processing Level	Radiometric Resolution
						* M	P		
3 April 1996	SPOT 3	HRVIR	0%	130	404	20 m	10 m	1A	8 bits
5 April 2009	SPOT 5	HRG	0%	130	404	10 m	5 m	1A	8 bits
2 April 2022	SPOT 6	NAOMI	0%	130	404	6 m	2.5 m	1A	16 bits

* M = multispectral, P = panchromatic.

**Table 2:** Description of different land cover classes in the study area.

Class	Description
Water bodies	This includes areas containing open bodies of water, such as brackish rivers, streams, flowing rivers, dams and natural ponds, as well as artificial ponds or canals.
Cropland	This includes all land used for agricultural activities. The area covers rain-fed and irrigated agriculture, and the types of cultivated crops grown in the study area include cereals, legumes and fruits.
Grassland	For the study area, the plants can be classified into lands predominately covered with grasses, forbs, shrubs and grassy areas.
Bare land	A land dominated by rock, unpaved road, eroded land and unusedLand; excavation, quarries and opencast mines; and areas of extensive overgrazing and woodcutting, etc.
Built-up	An impervious layer of land dominated by buildings, houses, paved road and huts, shopping centres and industrial, commercial and transportation facilities, including highways and major streets (tarred or gravel).

**Table 3:** Confusion matrix for classified images.

	1996		2009		2022	
Land Use Types	Producer	User	Producer	User	Producer	User
Cropland	75%	71%	75%	79%	83%	85%
Grassland	73%	71%	83%	85%	75%	79%
Bare Land	68%	73%	73%	71%	80%	82%
Built-up	78%	76%	85%	81%	85%	83%
Water Bodies	88%	90%	95%	95%	100%	93%
Overall accuracy	76%		82%			85%
Kappa coefficient	0.75		0.81			0.83

**Table 4:** (**A**) LULC area coverage, status and changes between 1996, 2009, and 2022. (**B**) Annual rate of change in land cover categories.

**(A)**												
**Land Use Types**	**Area**						**Change (Gain/Loss)**					
	**1996**		**2009**		**2022**		**1996–2009**		**2009–2022**		**1996–2022**	
	**Ha**	**%**	**Ha**	**%**	**Ha**	**%**	**Ha**	**%**	**Ha**	**%**	**Ha**	**%**
Built-up	1678	0.99	3877	2.30	7585	4.49	2199	1.30	3708	2.20	5907	3.48
Bare land	41,966	24.86	44,291	26.24	46,135	27.33	2325	1.38	1844	1.09	4169	2.47
Water bodies	5749	3.42	6164	3.65	8963	5.32	415	0.25	2799	1.66	3214	1.90
Grassland	69,626	41.25	75,816	44.92	63,197	37.44	6190	3.67	−12,619	−7.48	−6429	−3.81
Cropland	49,758	29.48	38,629	22.89	42,897	25.42	−11,129	−6.59	4268	2.53	−6861	−4.06
Total hectares	168,777	100	168,777	100	168,777	100						
**(B)**												
**Land Use Types**	**1996–2009**		**2009–2022**									
	**Ha**	**%**	**Ha**	**%**								
Built-up	169.2	0.1	285.23	0.17								
Bare land	178.8	0.11	141.85	0.08								
Water bodies	31.9	0.01	215.31	0.13								
Grassland	476.2	0.28	−970.69	−0.58								
Cropland	−856.1	−0.51	328.31	0.19								

The negative (−) indicates loss.

**Table 5:** Activity concentrations (mean) of ^210^Pb_ex_ and ^137^Cs in the surface and subsurface samples from the different land use areas.

		*Mean Mass Activity± SD* (Bq kg^−1^)	
Land Use Category	Radionuclides	Surface	Subsurface
Urban (built-up areas)	^210^Pbex	48 ± 7	3.21 ± 0.02
	^137^Cs	0.00	0
Agriculture (cropland)	^210^Pbex	10.01 ± 0.01	0
	^137^Cs	0.00	0
Mining (bare land)	^210^Pbex	10.01 ± 0.02	0
	^137^Cs	0.00	0
Resort/commercial (built-up areas)	^210^Pbex	140 ± 9	63 ± 32
	^137^Cs	0.00	0

**Table 6:** Results of the Shapiro–Wilk test for normality distribution.

	Surface		Subsurface	
Elements (mg/kg)	Values	*p*-Value	Values	*p*-Value
Be	0.684	0.007 *	0.968	0.831
B	0.803	0.108	0.907	0.467
Na	0.862	0.267	0.896	0.413
Mg	0.710	0.015 *	0.902	0.443
Al	0.861	0.262	0.879	0.335
P	0.710	0.015 *	0.838	0.189
K	0.824	0.152	0.727	0.023 *
Ca	0.802	0.107	0.965	0.813
Ti	0.930	0.595	0.873	0.311
V	0.924	0.559	0.984	0.924
Cr	0.637	0.002 *	0.898	0.419
Mn	0.630	0.001 *	0.873	0.309
Fe	0.838	0.188	0.949	0.710
Co	0.689	0.008 *	0.875	0.317
Ni	0.745	0.035 *	0.965	0.807
Cu	0.645	0.002 *	0.849	0.224
Zn	0.925	0.563	0.995	0.981
As	0.661	0.004 *	0.915	0.507
Se	0.767	0.055	0.891	0.386
Rb	0.779	0.069	0.977	0.886
Sr	0.755	0.043 *	0.868	0.290
Mo	0.640	0.002 *	0.963	0.797
Pd	0.864	0.275	0.940	0.657
Ag	0.784	0.076	0.942	0.668
Cd	0.687	0.008 *	0.730	0.024 *
Sb	0.812	0.125	0.798	0.099
Ba	0.772	0.060	0.939	0.650
Pt	0.896	0.412	0.904	0.451
Ti	0.665	0.004 *	0.892	0.393
Pb	0.696	0.010 *	0.940	0.655
Bi	0.659	0.003 *	0.982	0.914
Th	0.713	0.016 *	0.941	0.661
U	0.672	0.005 *	0.825	0.155

* Statistically significant at *p* > 0.05 and suggestive of a non-normal distribution.

**Table 7:** Geochemical fingerprints (mg/kg) that satisfied the pairwise multiple comparison tests.

Fingerprints	Surface Soil	Subsurface Soil
Mg	0.012 *	0.009 *
Ca	0.007 *	0.003 *
Al	0.003 *	0.001 *
Fe	0.002 *	0.013 *

* Significant at *p* > 0.05.

## Data Availability

Not applicable.
